# Supplementary material for: Are teachers missing the beat on students’ motor competence?
Source: PLoS One. 2026 Mar 16;21(3):e0344307. doi: 10.1371/journal.pone.0344307 (PMC12991265; doi:10.1371/journal.pone.0344307)
Supplement: Attachment — Submitted filename: PONE.docx [file pone.0344307.s002.docx]

PONE-D-25-64838

Are Teachers Missing the Beat on Students' Motor Competence?

**Abstract:** A brief reference to statistical methods is also provided.

**Introduction:** The introduction should refer more to the research background, more to the theoretical foundations, and also to the importance of the work, which should be after the necessity of the research. I did not see any explanation in this regard.

**Methods:** The entry criterion refers to the student's health, which was done by the instructor. Please indicate on what basis did the instructor assess the student's health? The validity and reliability of the tools are not mentioned.

**Discussion:** This section needs revision. You should first summarize the results of your work and then logically review your findings with the studies you mentioned in the introduction section. Examine the results of your work in more depth with previous studies. Also, better articulate the research that supports and contradicts your results.
