# Supplementary material for: Are teachers missing the beat on students’ motor competence?
Source: PLoS One. 2026 Mar 16;21(3):e0344307. doi: 10.1371/journal.pone.0344307 (PMC12991265; doi:10.1371/journal.pone.0344307)
Supplement: Attachment — Submitted filename: Review_AUTHORS-PONE-D-25-64838-Are Teachers....docx [file pone.0344307.s003.docx]

**General comment**

Understanding PE teachers’ perceptions of students’ motor competence is undoubtedly valuable, as these perceptions directly guide daily teaching–learning processes. The present study addresses an important and timely question. However, I have a central concern regarding the interpretation of the findings.

### Major Issues

Previous studies cited by the authors (e.g., Ruiz et al., 2001; Estevan et al., 2018) indicate that PE teachers can perceive students’ motor competence with certain degree of accuracy when it is referenced to **movement patterns or qualitative process measures**. In contrast, the present study relies on **kinematic variables (e.g., time, velocity)**, *which are less familiar in to PE teachers and in pedagogical contexts in general, and may require specific training for meaningful interpretation*.

Thus, the observed discrepancy between teachers’ perceptions and test outcomes may not reflect a limitation in teachers’ ability to assess motor competence per se, but rather a difficulty in interpreting **novel kinematic-based indicators**. This distinction is central to the manuscript and should have been clearly raised from the introduction but is only briefly acknowledged in the limitations.

Recent evidence suggests that, although kinematic variables may be strongly associated with performance outcomes, different kinematic indicators can lead to distinct interpretations of motor competence and would benefit from being examined alongside movement pattern analyses (e.g., Leite et al., 2025), which is the clearer reference we have for MC. This perspective may help contextualize the present findings and avoid overgeneralization regarding assessment accuracy in physical education.

Table 1 clarifies that teachers were asked to estimate performance outcomes expressed in specific quantitative variables (e.g., Kinematics, units, products), rather than to judge movement quality or execution patterns. This procedural detail strengthens the study’s transparency but also highlights a key interpretative issue: the observed discrepancies may reflect unfamiliarity with the metrics and units employed, rather than limited ability to perceive students’ motor competence per se; even with an ‘average child reference’.

Therefore, I recommend that the authors:

1. **Adopt more cautious language throughout the manuscript**, (from the abstract). Statements suggesting a general lack of assessment accuracy or the need for curricular adjustment should be attenuated and explicitly tied to the specific type of test and variables used, including “the level” of familiarity of the PE teachers with them.
2. **Clarify the pedagogical meaning of the kinematic variables employed**, emphasizing that accurate use of such measures likely requires targeted familiarization or training. This would strengthen the argument for professional development without implying deficiencies in teachers’ general assessment competence.
3. **Further articulate the value of the selected tests** by explaining how kinematic-based measures complement or are articulated to more traditional process-oriented assessments.

Overall, with clearer conceptual framing and moderated claims, this study has the potential to make a meaningful contribution to the literature on motor competence and its assessment in physical education.

**Minor issue:**

1. Check for typos and punctuations throughout the text.
2. Lines 95-100 conclude the introduction and point to the aim but do not specify it clearly as in the abstract. Check this when rewriting.
3. Line 104 says three schools and line 112 says 5. Check this.
4. Line 105 reads (2023–2024). I supose it is years. Specify it.
5. Lines 113. Specify how inclusion criteria were assessed.
6. Line 175. “outliers were checked and corrected or removed as needed.” Specify the criteria.
7. Lines 177-179. “Cohen's d ((mean 1 – mean 2)/pooled standard deviation) as the index of effect size (large - η2 > 0.8; moderate - η2 between 0.8 and 0.5; small η2 between 0.49 and 0.20; trivial - η2 < 0.2) (Cohen, 2013).” Consider rephrasing it.
8. Lines 181-183: “The estimation was considered accurate when the difference was lower than 5%. Differences of more than 5% were considered overestimations if the estimate exceeded performance”. Explain better the reason of this 5%.
9. Line 191. Gpower has a specific calculation for SPSS packeage. Was it considered? If so, state it in the sample session.
10. Line 214. I thinks it is “most teachers” instead of “more teacher”.
11. Figures. Enhance the quality of figure 1, and figure 2 is cut in the end (MC).
