# Supplementary material for: Are teachers missing the beat on students’ motor competence?
Source: PLoS One. 2026 Mar 16;21(3):e0344307. doi: 10.1371/journal.pone.0344307 (PMC12991265; doi:10.1371/journal.pone.0344307)
Supplement: Attachment — Submitted filename: Response to reviewers.docx [file pone.0344307.s006.docx]

Dear Editor,

We appreciate the new opportunity to improve the quality of our work. We followed the reviewers’ comments. Answers were provided in red in this file and the manuscript for better clarity.

Reviewer #3: General comment

1. Paragraph structure (Lines 103–120)

Lines 103-120 appear to contain more than one conceptual block and may benefit from being divided into two or three paragraphs. Revising this structure would improve clarity and readability. Check it out.

R: Done.

2. Conceptual clarity (Lines 112–113)

The sentence “Therefore, to the best of our knowledge, no other research has examined the accuracy of kinematic measurements based on PE teachers’ perceptions of their students’ MC.” appears conceptually imprecise.

Kinematic measurements are objective variables; what is being examined is the accuracy of teachers’ estimations relative to those objective measurements. I recommend revising the sentence to avoid potential misunderstanding.

Suggested revision: (Just a suggestion. See if it makes sense)

“To the best of our knowledge, no previous research has examined the accuracy of PE teachers’ estimations of students’ motor competence when compared to objective kinematic measurements.”

R: Thank you for the suggestion. Changes were performed accordingly.

3. Outlier handling not solved (Line 212)

The manuscript states that extreme statistical outliers were checked and corrected or removed as needed (Rodrigues et al., 2025). While removing outliers can be appropriate, the criteria used to identify and manage them are not clearly specified.

I recommend explicitly stating the criteria adopted (e.g., statistical thresholds, influence diagnostics, standard deviation cutoffs) and clarifying whether removal or correction affected the results. This would enhance transparency and reproducibility.

R: Thank you for the suggestion. Changes were performed accordingly, and we also added another investigation that supports our choice.

4. Justification of 5% tolerance not solved (Lines 219–222)

The manuscript indicates that a tolerance of 5% was adopted (Almeida et al., 2017). However, Almeida et al. (2017) does not appear to provide a clear justification for this specific threshold.

Please clarify the rationale for selecting 5% rather than another threshold (e.g. 3%, 7%, 10%) Was it a theoretical basis, empirical precedent, or practical reasoning? If the choice was based on researcher judgment, this should be explicitly acknowledged to allow critical appraisal.

R: We appreciate the feedback. We agree that Almeida et al. (2017) do not provide a formal theoretical explanation for using a 5% tolerance threshold. In our study, we chose the 5% criterion as a practical way to define meaningful differences in how people perceive things. This was done while also considering how difficult it is to estimate quantitative motor performance outcomes (e.g., time or velocity) in PE settings. We have updated the manuscript to more clearly state that this limit was based on practical considerations and is consistent with previous methods used in research on perceived versus actual competence. However, we also acknowledge that different limits could lead to slightly different classifications.

5. Figure quality not solved (Figures 1 and 2)

Figures 1 and 2 remain low in resolution and may not meet publication-quality standards. Please provide higher-resolution versions. Additionally, check for typos (e.g., “Locomtor” instead of “Locomotor” in Figure 1).

R: Thank you. The figures in the manuscript are high resolution, and we believe the reduced quality may be due to the PDF conversion or the viewing format. Additionally, we carefully reviewed the entire manuscript to identify and correct any typos or other writing inaccuracies. Thank you for bringing this to our attention.

6. Minor typos

Please review the manuscript thoroughly for minor typos (e.g., “inApril 2024”).

R: Done.
